# Translating Pathological Brain Activity Primers in Parkinson’s Disease Research

**DOI:** 10.34133/research.0183

**Published:** 2023-06-27

**Authors:** Daniela Mirzac, Svenja L. Kreis, Heiko J. Luhmann, Gabriel Gonzalez-Escamilla, Sergiu Groppa

**Affiliations:** ^1^ Movement Disorders and Neurostimulation, Department of Neurology, Focus Program Translational Neuroscience, Rhine Main Neuroscience Network, University Medical Center of the Johannes Gutenberg University Mainz, 55131 Mainz, Germany.; ^2^ Institute of Physiology, University Medical Center of the Johannes Gutenberg University Mainz, 55131 Mainz, Germany.

## Abstract

Translational experimental approaches that help us better trace Parkinson’s disease (PD) pathophysiological mechanisms leading to new therapeutic targets are urgently needed. In this article, we review recent experimental and clinical studies addressing abnormal neuronal activity and pathological network oscillations, as well as their underlying mechanisms and modulation. Our aim is to enhance our knowledge about the progression of Parkinson's disease pathology and the timing of its symptom’s manifestation. Here, we present mechanistic insights relevant for the generation of aberrant oscillatory activity within the cortico-basal ganglia circuits. We summarize recent achievements extrapolated from available PD animal models, discuss their advantages and limitations, debate on their differential applicability, and suggest approaches for transferring knowledge on disease pathology into future research and clinical applications.

## Introduction

Parkinson’s disease (PD) is the second most common neurodegenerative disorder, characterized by neuron loss along with the formation of intraneuronal inclusions across the central nervous system (CNS) called Lewy bodies. Alpha-synuclein is one of the main components of Lewy bodies, and its intraneuronal accumulation not only is related to cell death and neurodegeneration [1] but also disturbs the firing rate of dopaminergic neurons [2]. Moreover, neuronal cell loss leads to altered neurotransmitter signaling and dysfunction of excitation–inhibition balance, thus triggering abnormal patterns of action potentials, synaptic dysregulation, and pathologic activity in widespread brain circuits [3]. The cortico-subcortical circuits containing through the basal ganglia structures, thalamus, and the motor and premotor cortices create functional subregions known as the motor circuits [4]. These distinct functional loops show rhythmic oscillatory activity in different frequency bands, which are considered to be the hallmarks of PD [5]. Thus, understanding the mechanisms underlying these pathophysiological oscillations, as well as elucidating their interactions and modulation by different neurotransmitters, is of high relevance for the identification of early biomarkers and the advancement of innovative methods for targeting circuit dysfunction.

In this review, we will first explore the development of aberrant oscillatory activity within the cortico-basal ganglia system. Second, we will elaborate on the beta and gamma oscillations as electrophysiological hallmarks of PD. Third, we will expand on some of the relevant aspects of the molecular and genetic mechanisms involved in PD pathophysiology. We will then explore alpha-synuclein as a paramount component of the pathological cascade and continue with a discussion of the most common genes associated with PD: SNCA, LRRK2, PARK7, and PINK1. Further, we will elaborate on existing animal models of PD and their particular characteristics, in addition to showing their differential utility. Finally, we discuss the translational value of pathological electrophysiological activity studies as well as devising future perspectives.

## Brain Oscillation Fingerprints of Parkinson's Disease

Brain oscillations have emerged as a powerful tool to investigate the PD pathophysiology. The following section aims to provide an overview of the key findings in this field and how they can be utilized in forward and back translation.

**Fig. 1.**
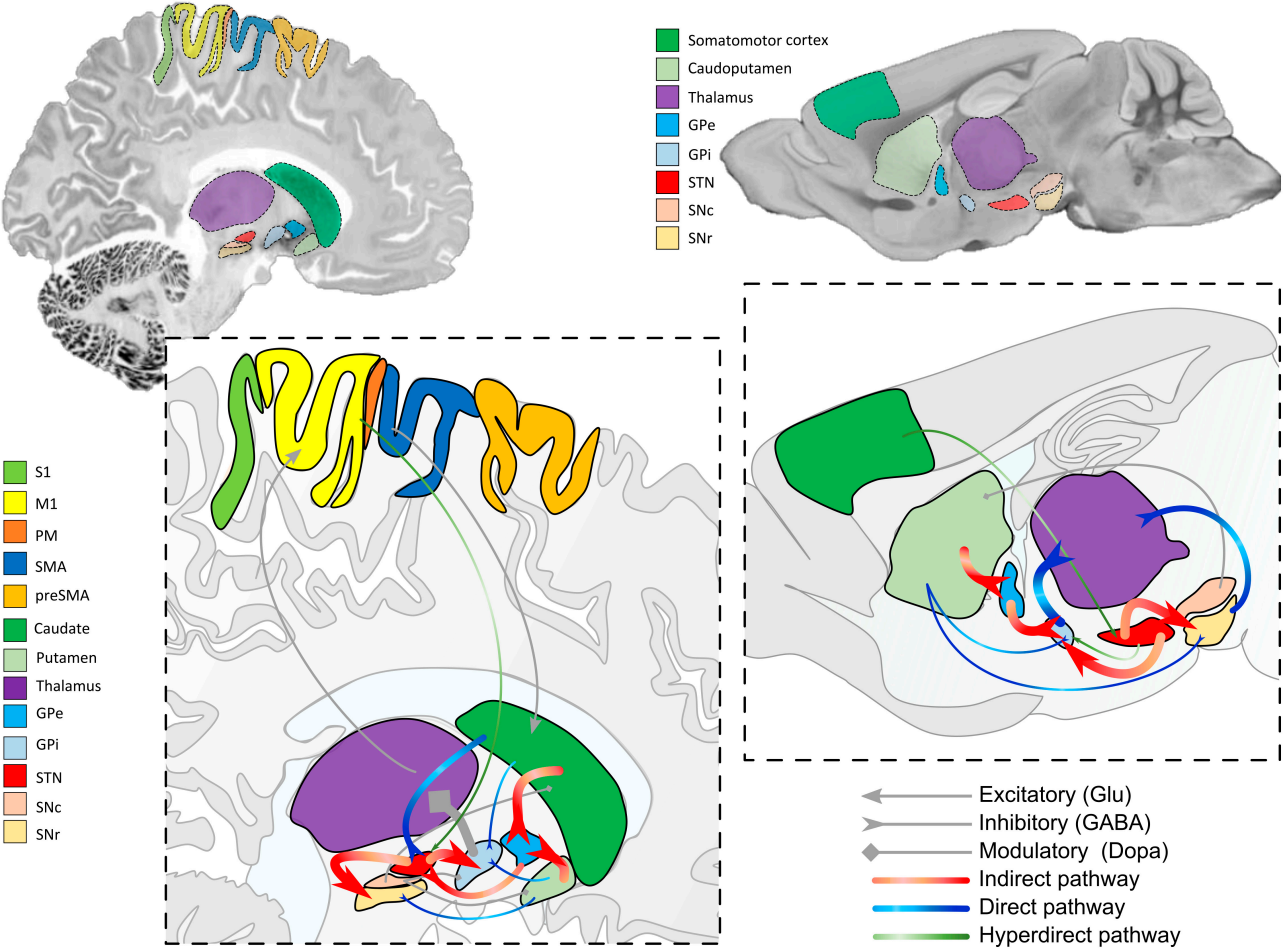
The cortico-basal ganglia network and motor pathways in Parkinson’s disease (PD) in human and mouse. Two-dimensional (2D) representation of the cortical and subcortical areas involved in motor control/performance in the human (left column) and mouse (right column) brain. For each brain, a depiction of the direct (blue arrows), indirect (red arrows), and hyperdirect (green arrows) pathway is given. The backdrop image for the human brain is reproduced from the BigBrain atlas, a near-cellular (20 μm) resolution 3D digitized model of a human brain [156]. The backdrop of the mouse model is a high-resolution magnetic resonance imaging (MRI) (15 μm) anatomic reference atlas of the C57BL/6J mouse [157] acquired at 16.4 T. Thick arrows represent increased activity, whereas thin arrows represent decreased activity, by PD. S1, primary somatosensory cortex; M1, primary motor cortex; PM, premotor cortex; SMA, supplementary motor area; preSMA, pre-supplementary motor area; GPe, globus pallidus pars externa; GPi, globus pallidus pars interna; STN, subthalamis nucleus; SNc, substantia nigra pars compacta; SNr, substantia nigra pars reticulata.

A robust way to study the basal ganglia network is via oscillatory activity patterns in the cortico-subcortical circuits that loop through the basal ganglia and motor cortices. We focus on particular nuclei of the basal ganglia, including the caudate and putamen (also known as the striatum), subthalamic nucleus (STN), and substantia nigra (SN). A comparative schema of the basal ganglia network for both humans and mouse models is depicted in Fig. 1. The anatomical circuit organization in mice resembles that in humans. In both species, the direct pathway originates from the striatum and projects to the SN pars reticulata and globus pallidus pars interna (GPi), whereas the indirect pathway originates from the striatum and projects to the globus pallidus pars externa (GPe), which in turn projects to the STN, which then projects to the SN pars reticulata and GPi [6]. However, some relevant macroscopic and microscopic anatomical differences have to be underlined. In mice, there is no structural separation within the dorsal striatum, whereas for humans, it is divided into the caudate nucleus and the putamen [6]. Additionally, the entopeduncular nucleus in mice serves as the functional homolog of GPi and is mostly located within the internal capsule, while the functional homolog of GPe is referred to as globus pallidus [6]. In PD, there is a selective loss of dopaminergic neurons in the SN pars compacta, leading to a reduction in excitatory inputs to the direct pathway and an increase in inhibitory inputs to the indirect pathway [7]. This results in an imbalance in activity between the direct and indirect pathways, with a relative decrease in direct pathway output and an increase in indirect pathway output [7]. Additionally, there is a decrease in synaptic plasticity [2] and aberrant oscillatory activity in the basal ganglia network [8].

Oscillatory activity is a rhythmic representation of neural synchronization across circuits [4] recorded by electrophysiological methods. Most commonly used are local field potentials (LFP) recorded with implanted electrodes within deep brain structures, and electroencephalographic data recorded with electroencephalography or electrocortical recordings at the cortical level.

**Fig. 2.**
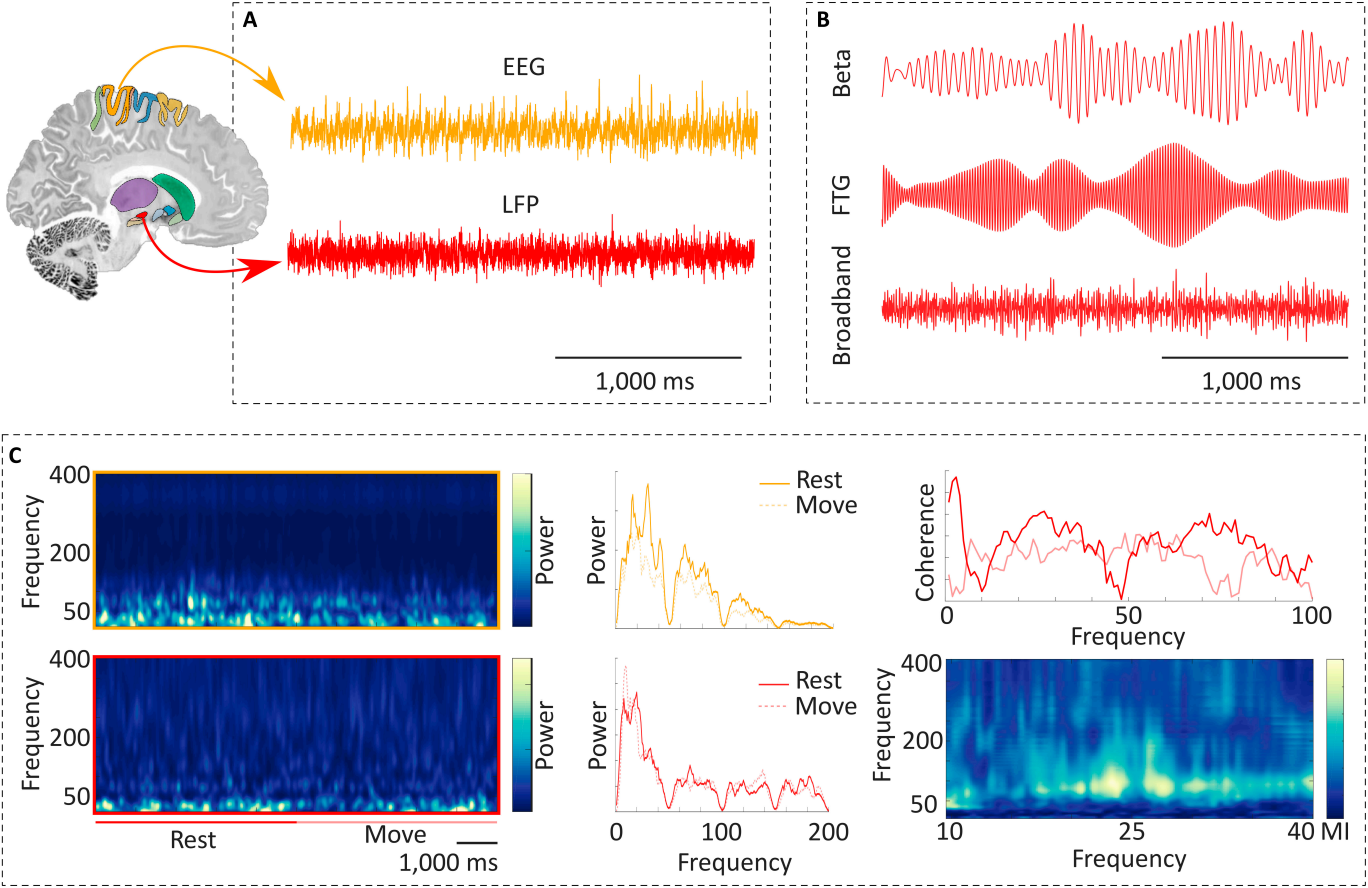
Oscillatory activity in a single patient with Parkinson’s disease (PD). (A) Raw signals at rest were recorded from a single patient with PD shortly after deep brain stimulation surgery. Electroencephalography (EEG) data were recorded from the electrode C3; concurrent local field potentials (LFP) data were recorded from the externalized electrodes implanted in the subthalamic nucleus (STN). (B) Same STN signal filtered at specific frequencies. Top: Filtered around the individual beta peak frequency (~20 Hz + −2 Hz). Middle: Filtered around 70 Hz, corresponding to finely tuned gamma oscillations. Bottom: Broadband gamma oscillatory activity between 35 and 200 Hz. (C) Left: Time–frequency dynamics for EEG and LFP data during the same rest interval followed by movement (spiral drawing). Beta power in the STN decreases with the beginning of the movement. Middle: Power spectra of the EEG and LFP channels (rest: continuous lines, movement: dashed lines). Both EEG and LFP present a spectral peak at around 20 Hz, which is reduced during movement execution. Right: Coherence (top) and cross-frequency coupling (bottom) between the EEG and LFP channels as markers of interregional connectivity. Increased cross-frequency coupling between beta frequencies (23 and 27 Hz) and broadband gamma frequencies (50 to 200 Hz). LFP, local field potentials; FTG, finely tuned gamma oscillations; MI, modulation index.

Electrophysiological studies analyzing LFP data from the STN in PD patients have reported abnormally synchronized oscillatory activity in the beta frequency band [13 to 35 Hz, with peak frequency between 16 and 20 Hz; see Fig. 2B (top) and Fig. 2C (middle panel)] as an electrophysiological hallmark of basal ganglia activity in PD [9–12]. This is congruent with previous studies showing that beta band oscillations and/or their coupling play a role in maintaining the excitation–inhibition equilibrium during movement [13]. Frequencies in the upper spectrum of the beta oscillation band (about 20 to 35 Hz), so-called high-beta activity, are more closely tied to cognitive functions like decision making and working memory [14]. Frequencies in the lower spectrum of the beta oscillation band (about 13 to 20 Hz) in the STN are more associated with motor symptoms like bradykinesia [15].

It was reported that motor cortex oscillatory activity in the beta band can trigger beta oscillations in the STN through the “hyper-direct pathway” [16]. Thus, elevated beta synchrony that exceeds the STN and affects the entire cortico-basal ganglia network is related to the severity of the motor symptoms (i.e., bradykinesia or rigidity) [4]. Therefore, STN beta activity is coupled to and preceded by beta and gamma band (about 50 to 200 Hz) cross-coupling exaggerated activity, and gamma activity peaks in the primary motor cortex (M1) [17]. Asymmetrical 2-way connections between mesial and lateral cortical regions and the STN within cortico-subcortical circuits were revealed by studying the LFP recordings from deep implanted electrodes alongside scalp electroencephalographic activity [18]. Namely, the study found that the cortex has a stronger impact on the STN in the beta band, with a notable reduction in beta band coupling from the mesial cortex to the STN during movement. In another study, LFP and electroencephalographic data showed that signal synchronization could be a crucial factor in separating physiological and pathological networks [19,20]. In this context, both the pharmacological treatment (i.e., dopamine medication) [21] and the invasive treatment [i.e., deep brain stimulation (DBS)] modulate the connectivity within the motor network [22]. STN DBS modulates synchrony within the STN, and selectively suppresses synchronization between the STN and mesial premotor regions, within the beta frequency range [23]. At the same time, it increases interconnectedness in the left and right motor cortex [22].

Within the basal ganglia nuclei, according to a biophysical model of striatal microcircuits, dopamine induces a switch between 2 network states: a low dopaminergic state and a high dopaminergic state [24]. A postulation for the activity in these states is that the low dopaminergic state is generally characterized by low gamma and high beta activity. The high dopaminergic state is characterized by alternating bursts of high-gamma and beta oscillations together with a delta (about 1 to 3 Hz)/theta (about 4 to 7 Hz) rhythm [24]. In regard to the oscillatory activity, STN beta power correlates well with parkinsonian symptoms (i.e., bradykinesia and rigidity) [7]. In a study on PD patients with STN-DBS, it was observed that there was a greater modulation of STN activity in the hemisphere with less dopaminergic innervation, particularly evident during movements made with the contralateral hand [25]. These findings suggest that there may be a relationship between striatal dopamine and the precise tuning of movement within the cortical-subcortical pathway. Additionally, the study highlights the unique processing of ipsilateral and contralateral movements within the cortical-basal ganglia motor system. However, further research is needed to confirm these preliminary results [25].

The patterning of beta band oscillations over time was reported to be relevant, beyond the average level of synchronization [26]. Within the motor network, beta oscillations can occur as brief, transient events or bursts [27]. In PD, beta activity in the basal ganglia is characterized by prolonged burst duration and lower burst rates compared to dystonia [28]. Single-neuron bursts that occur periodically have been found to encode pathological beta, as evidenced by the strong correlations in time–frequency and phase coupling between the bursting and LFP signals [29]. The amount of longer STN LFP bursts can be altered by medication (i.e., levodopa). The dopamine-depleted state (OFF medication) has been correlated with motor impairment [26], in particular bradykinesia and rigidity [10,30]. Meanwhile, administration of levodopa (dopamine ON state) leads to a decrease in the frequency and length of low-beta states; the greater the decrease, the better the improvement in motor abilities [31]. Duration of beta bursts in the STN has also been proposed as a possible biomarker for freezing of gait in PD [26,32].

Aberrant beta band activity is robust over time [33,34]. Recently, there has been an interest into the effect of the state of dopamine and movement on the long-range beta functional connections between the basal ganglia and lower motor neurons, utilizing both STN LFP and electromyogram recordings [35]. The study revealed that STN beta activity was most noticeable during the resting motor phase of PD patients and was attenuated in voluntary movement [35].

As mentioned above, LFP activity is found not only in beta bands but also in theta, delta, and gamma, all of which have different patterns. Thus, various spectral states in the STN have an effect on motor impairment, and changes in the balance of these states induced by levodopa can accurately predict clinical decline [31].

Unlike the akinetic effect associated with beta oscillations, gamma activity potentially relates to a prokinetic effect [27]. Whereas M1 presents deficient activation in PD [36], the STN beta frequency excessively coupled with gamma frequency within M1 [17] may demonstrate immoderate synchronization within the motor cortico-basal ganglia network [37]. For example, STN DBS in PD leads to an increase in gamma activity over frontal and parietal regions, which is negatively correlated with symptom relief [38]. Furthermore, clinically effective high-frequency STN stimulation modulates cross-frequency interactions and balances beta and gamma oscillations [8].

Of note, finely tuned gamma oscillations (see Fig. 2B) are a network oscillation in the cortico-basal ganglia-thalamic loop, which can be measured from the cortex or STN in patients taking dopaminergic medication and has been linked to dyskinesia [39]. The relationship between dyskinesia and oscillatory activity in the motor cortex and STN was investigated [40]. Consequently, dyskinesia is associated with a narrowband gamma oscillation in the motor cortex, ranging from 60 to 90 Hz, as well as a weaker oscillation in the STN [40]. In addition, the authors of the study found strong phase coherence between these regions [40]. Thus, the narrowband gamma oscillations (60 to 90 Hz) between cortex and subcortical modulators might be regarded as another possible electrophysiological biomarker [37].

A recent study reports a negative relation between reduced beta band and increased gamma band oscillations in M1, premotor cortex, supplementary motor area, STN, and cerebellum in clinically effective DBS [8]. It may be deduced that, pathologically, the status quo in PD is maintained via the increased beta oscillations, while gamma oscillations act independently, whereas a therapeutically accurate DBS intervention induces a negative association between these 2 oscillations, thereby reestablishing a balance between these brain activities supporting dynamic processing [37]. Furthermore, it has been reported that not only the beta oscillations and their interrelationship with gamma oscillations but also the waveform of beta band, which becomes less sharp in optimal DBS, are highly suggestive of decreased M1 input synchrony [41]. Also, a direct correlation between altered gamma oscillations and reduced long-term potentiation-like plasticity elicited by intermittent theta burst stimulation in the M1 was found [42]. It has also been reported that increased fronto-temporal gamma and parietal theta band oscillations are related to anxiety and cognitive impairment, while decreased temporal theta and fronto-parietal gamma band oscillations are associated with depression and advanced age in PD [43]. Theta and alpha oscillations have been shown to play a critical role not only in motor function but also in cognition [44]. Specifically, STN stimulation targeting peak baseline theta has been shown to improve overall verbal fluency [44]. Additionally, alterations in baseline alpha oscillations and task-dependent modulation of alpha and theta oscillations have been identified as potential neural markers of poor sequential working memory in PD [45]. Gait dysfunction in PD has been associated with reduced theta and increased beta power in frontal rhythms, which could be used as predictors of lower limb motor symptoms [46]. Oscillations in the low-beta and theta bands have been observed in the STN during freezing of gait in PD [47]. Elevated baseline alpha and theta oscillatory activity has been found in apathetic PD patients, which requires intervention in order to facilitate incentivized movement [48].

Delta oscillations have received far less attention in electrophysiological studies because these very low frequencies are particularly prone to movement artifacts and other non-oscillatory sources, which can be problematic in movement disorder patients. Therefore, their emergence, their propagation through the network, as well as their symptom correlation remain largely unknown. It has been reported that delta activity is typical in PD dementia [49] and that it might underlie the gambling behavior in PD [50].

The phenomenon of high-frequency oscillations (HFOs) above 200 Hz, often dismissed as spiking activity from only a small number of neurons, is reportedly generated by a large population of neurons [51]. In levodopa-treated PD patients, HFOs with distinct peaks at 319 ± 33  Hz have been observed in the STN [52]. These HFOs are modulated by dopamine, with a slowing of about 60 Hz in the dopamine-depleted state (265 ± 33 Hz) [53]. The amplitude of HFOs is positively correlated with dopamine tone and enhanced during voluntary movement, especially in the L-DOPA-treated state [52,53], similar to the modulation pattern of the 70- to 80-Hz HFOs observed in the STN of PD patients [54]. Phase–amplitude coupling of HFOs has also been reported by several studies, including modulation by the phase of beta oscillations and other frequencies below the beta band [51,53,55,56]. An oscillation with very similar characteristics has also been reported in the GPi [57].

Moreover, a prominent oscillatory peak centered at 200 to 300 Hz, which increased during movement, was identified as a biomarker for PD, with the magnitude of HFO modulation negatively correlated with bradykinesia in patients [58]. Therapeutic high-frequency stimulation (130 to 180 Hz) induces HFOs similar to those observed with pharmacological treatment, which can be used as a marker of successful recalibration of the dysfunctional circuit generating PD symptoms [56].

Similar modifications in brain oscillations have been reported in the cerebellum, particularly attenuated mid-cerebellar theta band power during cognitive and motor tasks [59]. Meanwhile, another study reported typical beta and gamma dynamics across the subcortical network, which includes the cerebellum [8]. Also, the pathways that interconnect the basal ganglia and cerebellum to the extensive cortical regions provide a structural basis alongside the functional one [60].

As previously mentioned, beta oscillations are better registered during rest and tend to be restored during task execution [35]. Both levodopa administration and adequate DBS treatment modulate LFP beta oscillations [61]. Even if most of the electrophysiological studies are performed in patients in later stages of the disease, enhanced beta band oscillations have been observed in the primary sensorimotor cortices of both hemispheres in the early stages as well [62]. As the disease progresses, these oscillations develop a hemispheric imbalance that is linked to movement execution [62]. Neurobiological factors such as these play a hand in modulating the oscillatory activity [63].

Overall, we could affirm that pathological electrophysiological activity is present within the basal ganglia, various cortical areas, and cerebellum of PD patients. The robust presence of the aforementioned oscillatory activity patterns and their interrelationship underline their electrophysiological hallmark status, which should be further investigated in a translational approach.

## Molecular Aspects of Parkinson's Disease

While brain oscillations are a powerful investigative tool, the molecular mechanisms underlying the disease remain elusive. In this section, we will delve into the molecular pathways involved in PD with the aim of integrating these findings with the oscillatory aspects discussed previously. We will explore mechanistic insights relevant for the generation of aberrant oscillatory activity within the cortico-basal ganglia circuits.

Alpha-synuclein is an important component of the pathological chain that results in neuronal death with consecutive dopamine reduction. Alpha-synuclein is physiologically found in its monomeric form in the cytosol of neurons, or attached to membranes and vesicular structures. Under pathological conditions, alpha-synuclein oligomers aggregate into fibrils to form Lewy bodies [64]. Approximately 90% of alpha-synuclein is phosphorylated at situs S129 within the C-terminal region [65]. Besides phosphorylated alpha-synuclein, Lewy bodies are composed of p62, ubiquitin, dysmorphic organelles, and lipid membranes [66]. These small aggregates are mainly neurotoxic forms [67] and are present in the degenerated areas of the PD brain [68]. Additionally, to alpha-synuclein, LRKK2 is also found in Lewy bodies, suggesting protein–protein interactions [69,70].

Although alpha-synuclein’s cytotoxicity was previously thought to be limited to a single neuron, recent evidence suggests that it can be released to the extracellular space, thus affecting neighboring neurons and glia cells [71]. Similar to a prion disease, different strains of pathological alpha-synuclein have been described: fibrils and ribbons, each with different seeding and propagation characteristics [72]. After being released into the cerebrospinal fluid [73], the misfolded protein spreads from affected to unaffected regions of the brain along anatomically connected networks [72]. Certain neuron populations are more vulnerable than others, thus resulting in specific degeneration of certain neuron populations while sparing others [72]. Indeed, only a small amount of preformed fibrils is sufficient to induce a pathologic conformation of the endogenous alpha-synuclein [74]. Of note, reactive oxygen species (ROS) production correlates with a lower concentration of alpha-synuclein aggregates, which suggests that both seeding and cellular stress might be required for the self-perpetuating recruitment [75].

LRRK2 and alpha-synuclein may contribute to the phosphorylation and buildup of tau protein [76]. Because the formation of alpha-synuclein inclusions is usually accompanied by neurodegeneration [75], it is key for both the generation of PD and the progression to more advanced stages of the disease [77]. Dependent on the direction of alpha-synuclein spread, 2 pathways could be described. The first pathway is characterized by nigrostriatal dopaminergic dysfunction prior to involvement of the autonomic nervous system [78]. The residence of alpha-synuclein aggregates in intestinal tissue of PD patients would suggest a second pathway (“gut first hypothesis”), where aggregates originate in the enteric or peripheral nervous system [77].

Alpha-synuclein impairs the physiological synaptic plasticity of the basal ganglia according to the following mechanism. Intra-striatal alpha-synuclein preformed fibrils impair the firing rate of the SN pars compacta dopaminergic neurons, while the discharge of putative GABA-ergic cells of the SN pars reticulata is unchanged [2]. Thus, the long-term potentiation and depression of synaptic plasticity is affected by disruption of nigrostriatal function [2]. As a consequence of the loss of dopamine signaling and network restructuring, a compensatory reorganization of the dopamine receptors arises across the nervous system [79]. Functional up-regulation of postsynaptic dopamine D2 receptors has been a consistent finding [79,80]. Specifically, the sustained increase in dopamine D2 receptor up-regulation in the striatum of PD patients contralateral to the primary motor symptoms suggests that the alterations in the receptors may be influenced by the underlying neurodegeneration [79]. Another recent study focused on the significance at the neural activity level that the functional up-regulation of postsynaptic dopamine D2 receptors has in PD patients [80]. Particularly, the authors found that PD patients showed increased molecular connectivity between both caudate/putamen and hyperactive parietal eye fields, as well as negative frontal–putamen functional–molecular association consistent with the reorganization shift [80]. These findings indicate a strong connection between functional activation and molecular-level synaptic changes, reflecting network reorganization in PD, in response to a progressive dopamine deficit in the fronto-striatal region [80]. Decreased levels of the postsynaptic dopamine D2 receptor combined with no differences of the striatal dopamine D1 receptor have also been reported [81]. Recent evidence highlights the therapeutical agonist activation of the dopamine D3 receptor interacting with the dopamine 1 receptor to reduce some side effects of the levodopa treatment [82].

Given that the motor cortex as well as basal ganglia have different functions regarding the movement, mapping the distribution across structures is very insightful. The posterior dorsomedial striatum neurons express either dopamine D1 or dopamine D2 receptors, which preferentially receive input from different structures [83]. In this study, the authors showed that neurons expressing dopamine D1 receptors communicate with secondary motor, secondary visual, and cingulate cortices, whereas neurons expressing dopamine D2 receptors receive input from primary motor and primary sensory cortices, as well as the thalamus. The same study showed that cortical structures predominantly display neurons expressing dopamine D1 receptors, which readily project to the striatum. In contrast, the thalamus and SN pars compacta display neurons expressing dopamine D2 receptors that network with the same type of neurons from the striatum [83]. It has been reported that dopamine receptors could also directly regulate the activity of other neurotransmitters [84]. This in turn emphasizes the great impact dopamine receptors have on the entire brain, and how variance in expression or interaction could affect the pathology as well as the clinical presentation of PD.

**Fig. 3.**
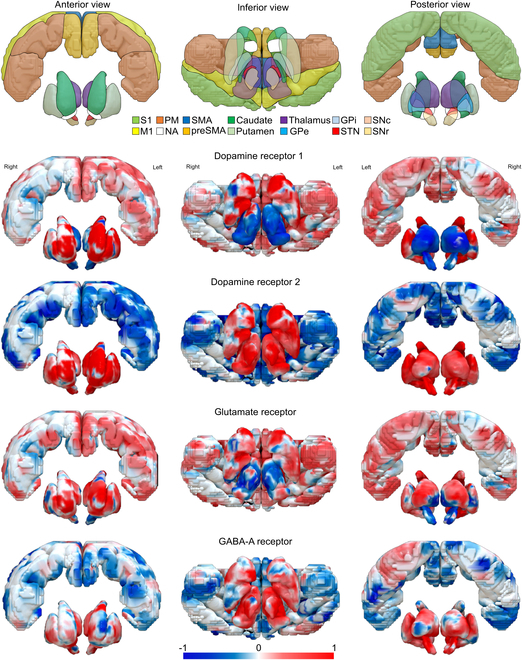
Distribution of dopamine D1, D2, glutamate, and GABA-A receptors in the cortico-basal ganglia. From top to bottom: 3D representation of the cortico-basal system, including a segmentation of the motor cortex according to the Human Motor Area Template (HMAT) [158]. Subcortical structures were manually delineated based on the multicontrast PD25 atlas, a PD population-specific template atlas [159]. Average gene expression profiles for dopamine receptors 1 and 2, as well as glutamate and GABA-A receptors derived from mRNA expression profiles derived from 6 adult human brain tissue samples mapped to Montreal Neurological Institute (MNI) coordinates as reported in the Adult Human Brain Atlas (AHBA) [160]. Within the basal ganglia itself, the caudate, putamen, and globus pallidum have the highest gene expression (red scale) of dopamine 1 receptors, whereas expression of these receptors is more distributed and less accentuated in the motor areas. The distribution pattern of dopamine 2 receptors includes higher expression in the SN, STN, and thalamus with down-regulated expression (blue colors) in the cortex. The gene expression for the glutamate receptor is similar to that of dopamine receptor 1 in the cortex but follows a different pattern in the subcortical nuclei, while the same holds true for GABA-A and dopamine receptor 2. Of note, AHBA has a broader coverage of the left hemisphere, and thus, no inferences about laterality can be reliably made for the gene expression based on these data.

Exemplary distribution of the dopamine receptors (as from gene expression patterns) across the basal ganglia and the cortex structures directly involved in PD is depicted in Fig. 3. Dopamine D1 and D2 receptors, as well as glutamate and GABA-A receptors, are widely distributed throughout the cortico-basal ganglia circuit. The striatum, which is the main input structure of the basal ganglia, has the highest concentration of dopamine receptors, with dopamine D2 receptors being more abundant than dopamine D1 receptors. In contrast, the cerebral cortex has a high concentration of both glutamate receptors and GABA-A receptors, which are involved in modulating cortical excitability and inhibiting unwanted movements. The STN, which receives excitatory input from the cortex and inhibitory input from the GPi, has a high concentration of glutamate receptors, while the GPi has a high concentration of GABA-A receptors. Although it has been shown that dopamine receptors are in decline over time, age seems not to have an impact on dopamine synthesis capacity [85]. Further, an up-regulation of postsynaptic dopamine D2 receptor in patients with mild to moderate PD [80], as well as in advanced stages has been described [79,81]. In early-stage PD patients, an increase in dopamine receptor density in the striatum has been observed, although using nonselective ligands for dopamine D2 and D3 receptors [86]. Unfortunately, no study exists comparing gene expression of dopamine receptors across the PD spectrum. This would shed light about the association between receptor expression and pathological development even at the preclinical states of the disease.

The reason for dopamine D3 receptor’s elevation in early-stage PD may be due to its promotion of T cell activation and induction of neuroinflammation, which could be an underlying factor in the role that dopamine D3 receptor’s activation plays in PD pathogenesis. Epigenetic mouse studies indicate a possible association between decreased levels of dopamine D2 receptor and susceptibility to specific inflammatory markers [87]. The anti-inflammatory properties of dopamine receptor stimulation have gained attention due to the influence of dopamine depletion and therapeutic dopamine replacement on the functions of astrocytes and microglia in PD [88]. It has been proposed that the early up-regulation of dopamine D2 receptors during development may lead to persistent alterations in striatal circuitry, resulting in long-term effects on motivated behavior that can be mitigated by reducing dopamine D2 receptor expression [89]. Other studies have reported the bidirectional regulation of striatal enkephalin expression by dopamine dopamine D2 receptors and the modulation of extracellular neurotransmitter levels in dorsolateral striatum via dopamine dopamine D2 receptor signaling by astrocytes [90]. Moreover, the disruption of dopamine receptor D1 localization to primary cilia impairs signaling in striatal neurons, and phosphodiesterase 10A (PDE10A) has been identified as a regulator of dopamine agonist-induced gene expression in the striatum [91].

## Animal Models of Parkinson's Disease

Considering the pathophysiological hallmarks of PD, mentioned in the previous section, an animal model should encompass as many of the following characteristics as possible [92]: (a) physiological number and function of dopamine neurons at birth with subsequent selective and gradual loss, exceeding 50% readily detectable, in adulthood; (b) easily visible motor impairments; (c) formation of Lewy bodies; (d) single mutation that spreads effectively in genetic-based models; and (e) short disease course.

Although mice are regarded as inferior on an evolutionary scale of species, and this review includes other species also (i.e., nonhuman primates and rats), mice are widely used for generating new disease models and we will focus mostly on them. In mice, motor deficits can be correlated with the nigrostriatal dopaminergic degeneration as identified with histological methods [93]. Phenotypical and histological data recorded in PD models resemble those obtained in PD patients; this consequently supports the use of models in electrophysiological studies. Pathophysiological network oscillations described in mouse models discussed below not only replicate human findings but also give deeper insights into their underlying mechanisms and modulation.

Since idiopathic parkinsonism is heterogeneous in its etiology, disease models have been developed using 2 approaches: a neurotoxin-based approach to account for the environmental factors and a genetic-based one examining the genetic factors [92]. New models combining both genetic and immune approaches are being developed to study the role of the immune system in the development of PD [93]. We will discuss in detail the neurotoxin models first, followed by the genetic models.

### Neurotoxin-based models

Neurotoxin-based models cause a rapid decline of nigrostriatal dopaminergic neurons, caused usually by oxidative stress, which mimics sporadic PD [93]. These models are based on toxins, including 6-hydroxydopamine (6-OHDA), 1-methyl-4-phenyl-1,2,3,6-tetrahydropyridine (MPTP), and others. Systemic toxins (e.g., MPTP) are usually given to primates, whereas inoculable toxins (e.g., 6-OHDA) are injected directly into specific sites, i.e., SN and striatum, of rodents [94]. Reversible pharmacological treatments can disrupt dopamine signaling, by altering its packaging, release from presynaptic terminal, or ability to activate dopaminergic receptors [95].

Most of these toxins have a similar mechanism of action. The substance oxidizes to its active form, then enters the cell via the dopamine transporter or by diffusion, and concentrates in mitochondria where it inhibits respiratory chain complex I. On the one hand, this will reduce adenosine triphosphate production; on the other hand, ROS production is increased [96]. Depending on the dose of the toxin, it could boost either necrotic or apoptotic cell death [92]. Although these neurotoxin-based models lack formation of Lewy bodies, they largely reproduce the PD pathophysiology in humans and allow a quantifiable assessment of the electrophysiological activity across the brain, independent of the toxin.

#### 6-OHDA

The 6-OHDA neurotoxin is often used to create specific lesions in the nigrostriatal dopaminergic system, resulting in behavioral, histological, and electrophysiological changes that closely resemble PD.

The correlation between beta activity and symptom severity has long been established [97]. Still, it is important to mention that only STN, and not cortical beta oscillations, is correlated with the histological markers of the dopaminergic neurodegeneration in SN [98]. This finding is especially noteworthy because it argues in favor of this model, because of its correlation with human data. Not only STN beta oscillations were identified before any M1 activity, but also the percentage of long beta bursts increased over time [98]. Moreover, the phase amplitude coupling between theta and gamma activity in the striatum suggests that this parameter could be used as a biomarker of disrupted neural mechanisms in PD [99]. Hypersynchronized oscillatory activity in the beta band and decreased firing rate of striatum neurons were reported in a 6-OHDA mouse model during movement [100].

In a recent study on mice, conversions in the LFP oscillations of the striatum were assessed at different time points after the 6-OHDA inoculation [99]. Increased power in the theta, alpha, beta, and low gamma bands 15 days after inoculation was reported. Additionally, the study included levodopa treatment starting in the 15th day, which significantly reversed the activity in the beta and low gamma bands to control levels. On day 19 after inoculation, which was also the fifth day of treatment, increased delta activity was recorded, in addition to the low gamma band activity being reversed to control levels. It is unclear whether the delta frequency oscillations and treatment irresponsive of the beta activity could be a sign of disease advancement [99].

In an awake mouse model of dopamine depletion, it was demonstrated that delta oscillations in SN pars reticulata are a strong indicator of dopamine neuron loss and akinesia [101]. These oscillations, despite being previously disregarded as motor or anesthesia artifacts, may arise from diminished dopamine 2 receptor activation and precede motor cortex oscillations with a 100- to 300-ms lag [101].

#### MPTP

One of the most characterized neurotoxin-based models of PD is the MPTP model. This neurotoxin is highly lipophilic and suitable to induce bilateral lesions. However, this model has some disadvantages: SN pars compacta neurons are more vulnerable than neurons from the ventral tegmental area [92]; pronounced degeneration of noradrenergic neurons in the locus coeruleus [102]; the striatum depletion [103] requires chronic injection with low MPTP doses; and greater loss of dopamine terminals in the putamen [92]. Given the characteristic of this toxin, it is not well suited for rodents in general; therefore, it is used primarily for nonhuman primate studies.

It is widely known that MPTP correlates with increased alpha-synuclein levels [104]. Furthermore, alpha-synuclein oligomerization, described in the MPTP model, correlates with its phosphorylation, which is accompanied by alterations in its regulatory enzymes [105]. The relationship between phosphorylated alpha-synuclein and hyperphosphorylated tau, which is a pathological feature of PD, is not particularly clear in the MPTP model [1].

Alpha and low-beta oscillatory activities in the striatum that correlated with cortical and GPi oscillations were reported in a nonhuman primate MPTP model [106]. The previous findings have been proven somewhat controversial by another study also on nonhuman primates, which registered beta band oscillations to resonate through the STN, not the striatum [107]. Moreover, it was demonstrated on an MPTP model that beta activity, though not necessarily always pathological, is more prevalent in parkinsonism and that longer episodes of beta activity are a biomarker of abnormal neural activity [107,108].

Transcranial ultrasound stimulation has been proposed to normalize the network activity in an MPTP mouse PD model [109]. The study showed that after stimulation, there was a significant decrease in beta activity, the strength of phase amplitude coupling between beta and high gamma (around 55 to 100 Hz) and between beta and ripple (around 100 to 200 Hz) [109]. Concordantly with what has been done on humans, a study on nonhuman MPTP primates showed that under normal conditions, HFOs and their modulation were not significant; however, they emerged in PD [58]. In PD patients, movement-modulated gamma oscillations in the GPi, with a peak at 200 to 300 Hz, are a robust pathological aspect [58]. This finding supports the hypothesis that HFOs in the GPi are pathophysiological features of PD and warrants further investigation into their role(s) in motor control [58].

### Genetic-based models

Another etiological factor for developing rare or familial types of PD is the genetic factor. The development of genetic models of PD involves overexpressing various genetic mutations, for example, in alpha-synuclein, LRRK2, PARK2, PARK7, and PINK1. Such mutations allow to study the role of these particular genes in the pathology of PD [93].

#### Alpha-synuclein

Alpha-synuclein is a protein encoded in the SNCA gene that is responsible for familial autosomal dominant PD [110]. Since mice that lack the alpha-synuclein gene are viable and show no damage in the brain or its dopaminergic neurons, it is more likely to assume that overexpression mutations will cause PD [111]. Well-known mutations of alpha-synuclein include substitutions (A53T, A30P, and E46K), duplication, and triplication [112].

Studies on transgenic mouse models have shown progressive accumulation of alpha-synuclein in various brain regions, but with varied results in terms of inclusion formation and dopamine cell loss [113]. Some models showed formation of alpha-synuclein inclusions in the hippocampus, SN, and neocortex, but did not exhibit the fibrillar structure of Lewy bodies [114]. Others demonstrated progressive accumulation of alpha-synuclein and ubiquitin inclusions in the same brain regions through the use of different promoters [114]. These inclusions were of a fine granular material, and electron microscopy described no fibrillar aggregates similar to Lewy bodies. Furthermore, loss of dopamine terminals was assessed by immunocytochemistry and decreased tyrosine hydroxylase protein levels were reported. To overcome the fact that the aforementioned models do not develop abundant dopaminergic cell loss, a genome-based bacterial artificial chromosome transgenic mouse model was developed, which reports systemic alpha-synucleinopathy [115].

Transgenic mouse models have not been successful in reproducing the disease in a reasonable time frame, with appropriate electrophysiological activity and histology presentation. Therefore, a model of viral vector-mediated overexpression of alpha-synuclein was created to study the neurodegenerative process and the extent of SN dopaminergic neuronal loss. This study showed significant and dose-dependent alpha-synucleinopathy, with substantial loss of dopaminergic neurons in the SN, reaching a maximum of 82% after 8 weeks, in a recombinant adeno-associated viral vector serotype 2/7 (rAAV2/7) mouse model [116]. However, no electrophysiological investigations to describe particularities of the oscillatory activity were performed by the authors. However, a similar mouse model reported increased beta and gamma spectral power in the primary motor area with higher beta band connectivity between primary motor and premotor mouse brain area homologs, which correlates well with the human studies [117]. A secondary finding was beta- and gamma-mediated flow of information from premotor to motor mouse brain area homologs, also replicating the human data [117].

Subsequently, in rats, in a similar alpha-synuclein model created via viral vector injection in the right SN, LFP oscillations in the beta band were not identified [118]. The authors further reported beta and high-frequency oscillations that emerged only during severe parkinsonian conditions and were unresponsive to levodopa or spinal cord stimulation [118]. It has also been reported on mice that instead of an increased beta activity, a small decrease was found along with subtle adjustments in neurons’ firing; this suggests that the pathophysiology in genetic mice could be different than that in toxic dopamine depletion [119].

In contradiction, it was also reported that STN increased burst firing in a viral overexpression of an alpha-synuclein rat model [120]. Moreover, age-dependent impairment of mitochondrial function and gamma frequency (and greater sensitivity to rotenone) associated with the abnormal expression of alpha-synuclein in mice has also been reported [121].

The alpha-synuclein preformed fibril injection model involves injecting preformed fibrils of alpha-synuclein oligomers into loci of interest in the brains of rodents [122]. Models utilizing preformed fibrils of alpha-synuclein induce a range of pathogenic changes, including protein aggregation and neuronal death [123]. These changes arise from the progressive aggregation, which is triggered by a single injection of a small amount of aggregates made from the homologous protein [122]. In mouse brain slices exposed to alpha-synuclein oligomers, electrophysiological recordings revealed impaired synaptic transmission and long-term potentiation in the hippocampus [124]. Similarly, another study showed dysfunction in synaptic transmission and plasticity, as well as long-term potentiation impairment in the striatal medium spiny neurons on electrophysiology recordings [125]. Further investigation revealed that alterations in the molecular composition of *N*-methyl-d-aspartic acid receptors containing Glu-N2A subunits were responsible for this phenomenon [125].

#### LRRK2

Mutations in the LRRK2 gene are associated with familial autosomal dominant PD [126]. Two of the most common mutations are G2019S and R1441C/G [127]. It has been suggested that the mutant G2019S form of the LRRK2 serine-threonine kinase protein may directly interact with and phosphorylate alpha-synuclein, leading to the formation of alpha-synuclein aggregates similar to Lewy bodies [128].

In a bacterial artificial chromosome transgenic mouse model with the LRRK2-R1441G mutation, the authors showed axonal pathology in the striatum, without the presence of dopamine cell loss and alpha-synuclein aggregations [129]. Another study using a herpes simplex virus viral vector LRRK2-G2019S mouse model showed a 50% degeneration of dopaminergic neurons in the SN [130]. To better understand the relationship between alpha-synuclein and LRRK2, double transgenic mice may be utilized [131,132].

An increase in STN burst firing in a mouse model of alpha-synuclein viral overexpression was reported [120]. This aberrant firing pattern was reversed through genetic LRRK2 ablation or LRRK2 kinase activity inhibition [120].

#### Parkin

Mutations in the PARK2 gene manifesting the loss of function account for autosomal-recessive early-onset PD [133]. Parkin is an ubiquitin E3 ligase that regulates a variety of cellular processes [134]. More specifically, Parkin (and other proteins like PARK7 and PINK1) participates in mitophagy (i.e., clearance of impaired mitochondria through mitophagy) [135,136].

Elevated neuron activity levels, with reduced baseline firing rates of transgenic striatal neurons and their disrupted microcircuitry, were reported in a genetic Parkin mouse model [137]. Likewise, striatum and GPi LFP displayed elevated beta oscillation phase coupled to the cortex [137]. Therefore, it was concluded that the loss of Parkin function leads to increased synchronized cortico-striatal oscillations in beta band and intra-striatal reconfiguration of interneuronal circuits, which will then predispose to an imbalanced striatal outflow, a finding that is typical in PD [137].

In A53T alpha-synuclein-overexpressing mouse models, alpha-synuclein accumulates in the mitochondria, causing increased mitophagy and neuronal death, which is mediated through a Parkin-dependent pathway [138]. No evidence of either nigrostriatal, cognitive, or noradrenergic dysfunction was reported in a PARK2 exon2 deletion model [139]. In a PARK2 exon3 deletion mouse model, behavioral impairments were noted along with impaired hippocampal long-term potentiation, which resulted in memory deficits, without motor involvement [140]. Therefore, this model would be more suitable to study the early stages of PD. Despite the use of these models, it is important to recognize that they have limitations and only poorly imitate human PD symptoms [138]. This is evident in the lack of spontaneous degradation of the nigrostriatal pathway and increased vulnerability of dopaminergic neurons [140].

### Pharmacological model

Reserpine irreversibly inhibits vesicular monoamine transporter 2, leading to loss of storage capacity and depletion of monoamines, including noradrenaline, serotonin, and dopamine [141]. This inhibition also results in the growth of neurotoxic dopaminergic oxidation by-products such as dopamine quinones, which generate ROS via molecular oxygen [142]. Additionally, enzymatic breakdown of dopamine through monoamine oxidase further increases ROS formation [143]. When the production of ROS exceeds the antioxidant system’s ability to neutralize them, oxidative damage occurs as described with the neurotoxic model.

Systemic administration of reserpine depletes dopamine stores at nerve terminals and induces a hypokinetic state in rodents due to the loss of dopamine storage capacity in intracellular vesicles [141]. Repeated reserpine treatment induces serotoninergic dysfunction, reduces serotonin circuitry in corticolimbic areas, and lowers serotonin immunoreactivity in hippocampal areas (CA1 and CA3) [144]. Enhanced beta-oscillations, a prominent feature of the pathophysiology in the cortico-basal ganglia of reserpine and 6-OHDA models of PD, show substantial differences in peak frequency between the 2 groups [145]. In the 6-OHDA group, a beta peak was also found in M1 at 18 Hz, which was not detected in the cortex of rats treated with reserpine [145]. Furthermore, both the acute reserpine model and chronic 6-OHDA model have exhibited enhanced beta oscillations in the basal ganglia during the activated network state [146]. Nevertheless, studies have produced ambiguous findings concerning changes in beta power in animals treated with a D1/D2 receptor blocker [147]. While some studies have reported an increase in beta band oscillations under acute pharmacologic dopamine blockade [148,149], others have not observed such changes [150,151].

The key oscillatory findings in animal studies have been summarized in Table.

**Table.** Findings about oscillatory brain activity in animal models.

Region	Species and disease model details	Oscillatory brain activity	Reference
Striatum	ICR mice injected with 6-OHDA in the left striatum	Increased power of theta, alpha, beta, and low gamma bands; Increased theta–gamma coupling in the striatum;	[99]
	C57BL/6J mice injected with 6-OHDA in the dorsolateral striatum	Elevated beta oscillations;	[100]
Medial forebrain bundle	Wistar rats, 6-OHDA injected into the medial forebrain bundle	Increase in beta oscillations in STN preceded increase in beta oscillations in motor cortex; STN beta power correlated with motor impairment; Only STN but not cortical beta power correlated with the histological markers of dopaminergic neurodegeneration; Higher percentage of long beta bursts;	[98]
	Sprague–Dawley (Harlan) rats, 6-OHDA injected into the medial forebrain bundle	Enhanced low-frequency band oscillatory activity and synchronization both within the SN pars reticulata or STN and with the cerebral cortex;	[101]
	Male Wistar rats; 6-OHDA injected into the medial forebrain bundle, reserpine systemically	Enhanced beta oscillations in the basal ganglia of both animal models; Reserpine-treated animals showed no involvement of primary motor cortex, compared to 6-OHDA model; Both models exhibited elevated beta power; in the 6-OHDA model, mean peak frequency was located in the low-beta range (17 Hz), while in the reserpine model peak was centered at higher beta frequencies (27 Hz); Elevated beta coherence between primary motor cortex and basal ganglia in both models;	[146]
Substantia nigra	Male Sprague–Dawley rats, injected with AAV vectors encoding human wild-type alpha-synuclein in the right SN	Increased beta and high-frequency (>90 Hz) oscillations;	[118]
Systemic injection (targets unexclusively dopaminergic neurons) (whole brain)	C57BL/6J mice injected with MPTP	Increased beta (13–30 Hz) power; Increased phase–amplitude coupling between the beta and high-gamma (55–100 Hz) bands and between the beta and ripple (100–200 Hz) bands;	[109]
	Female rhesus monkeys (*Macaca mulatta*), male cynomolgus monkeys (*Macaca fascicularis*), injections of MPTP	Alpha (8–13 Hz) and low-beta (13–20 Hz) band oscillations found in the striatum, motor cortex, and GP; Both alpha and low-beta frequency band oscillations of the striatum were highly coherent with the cortical and GP oscillations;	[106]
	Female African green monkeys (*Cercopithecus aethiops aethiops*), injections of MPTP	Pathological basal ganglia beta oscillations consisted of synchronized time-limited episodes, rather than a continuous stretch, of beta oscillatory activity; Beta oscillations resonate across the basal ganglia network through the STN, not only the striatum; The duration of basal ganglia beta episodes is more impacted than their magnitude; Prolonged STN beta episodes	[107,108]
	Female rhesus monkeys (*Macaca mulatta*), injections of MPTP	Prominent oscillatory peak centered at 200–300 Hz that increased during movement;	[58]
Dopaminergic neurons (whole brain)	Alpha-synuclein-overexpressing mice, Masliah line 61	Decreased beta oscillations in the desynchronized state in SN pars reticulata, the ventromedial nucleus, and the primary motor cortex;	[119]
	Alpha-synuclein A30P-overexpressing mice	Age-dependent reduction in gamma power (20–80 Hz)	[121]
	Parkin knockout mice	Enhanced and phase-shifted phase coupling to slow (1–3 Hz) cortical population oscillations in the entire transgenic striatal microcircuit (motor cortex, striatum, and GP); Amplified beta oscillations (~22 Hz) in LFP from striatum and GP, phase-coupled to cortex	[137]
	Dopamine transporter knockout (C57BL/6J DAT-KO) mice, inducible and reversible pharmacogenetic approach (AMPT)	Corticostriatal activity asynchronous in hyperdopaminergic phase, while synchronicity increased during dopamine depletion phase; Neuronal oscillations and ensemble activity coordination within and between cortex and striatum changed rapidly between phases;	[148]

6-OHDA, 6-hydroxydopamine; MPTP, 1-methyl-4-phenyl-1,2,3,6-tetrahydropyridine; SN, substantia nigra; STN, subthalamic nucleus; GP, globus pallidus; LFP, local field potential

## Challenges and Future Perspectives

As the field of PD research continues to evolve, new challenges and opportunities emerge. In this section, we will discuss the major challenges the field faces, including the potential for integrating molecular and oscillatory aspects of the disease to develop new, targeted models. Finally, we will outline the future perspectives of PD translational research, highlighting key areas of focus.

PD animal models, either neurotoxic or genetic based, have advantages and limitations, which have been discussed at length in previous sections. The results obtained depend greatly on the model selected. On one side, systemic toxins (e.g., MPTP) affect systems beyond dopamine that may not be perturbed under pathological circumstances, and toxins that require a targeted injection (e.g., 6-OHDA) could induce a postoperative inflammatory state [94]. On the other side, genetic models have the advantage of depleting the brain of dopamine much more gradually, which better mimics the course of PD. A disadvantage of the modest neuronal damage is that the genetic models have yet to show the full spectrum of electrophysiological activity.

Poorer behavioral and clinical presentation in the genetic models compared to the toxic models discouraged initial electrophysiological studies. Nevertheless, some researchers have already shown some oscillatory pathological activity in genetic models, albeit scarcer than the one described with neurotoxic models. Consistent beta and gamma band perturbations are therefore identified. Promisingly, histological results that show quantifiable and prominent dopaminergic neuron loss [116] give the necessary confidence and await future electrophysiological studies on genetic models.

In order to bypass the disadvantages of each individual model, one potential solution to this issue is to use a combination of 2 models that replicate complimentary aspects of the disease in order to create a model of PD that better mirrors the pathology, the dynamics of the disease, and the activation states. Respectively, a transgenic mouse model that displays electrophysiological alterations, as well as motor and nonmotor deficits could be further developed to better bridge the gap between the behavioral and histological test results and the electrophysiological findings. To this end, the neurotoxins available are quite harsh and unspecific and defeat the purpose of a targeted and gradual depletion. Either the administration of a pharmacological substance like reserpine or the overexpression of alpha-synuclein (viral induced or via preformed fibrils) could be regarded. When combining two models, one has to consider the particularities of the mechanisms involved and account for possible interactions.

As previously mentioned, there is a distinct expression of the pathology in specific brain regions and circuits that are consistently affected in both human patients and mouse models of the disease. These regions and circuits include the SN, the striatum, and the cortico-striatal pathways [6]. The choice of brain region targeted in a PD model can have a significant impact on the symptoms observed and the underlying pathological mechanisms. For example, if a model targets the striatum rather than the SN pars compacta, the symptoms observed may be different, as the striatum is a key regulator of movement control and also receives inputs from other neurotransmitter systems, such as the glutamatergic and cholinergic systems [100]. A model targeting the cholinergic system in the basal forebrain may produce cognitive deficits similar to those seen in PD patients, as this system is known to be affected in dementia associated with PD [49]. However, as we discussed above regarding the nonrandom propagation properties of alpha-synuclein, future models have to account for the gap between the inoculation site and the impact of the spread of the pathological alterations. Nevertheless, given that PD is a heterogenetic disease, having a variety of models targeted toward specific research questions is more appropriate than having a trade-off between genetic, neurotoxic, and environmental aspects.

Regardless of which model is used, we are in need of more robust and replicable electrophysiological data of the beta activity characteristics addressing long and short bursts, as well as their correlation to motor function, dopaminergic tone, and disease progression. Findings of balanced beta in favor of gamma activity coupled with dopamine repletion or electrical stimulation, which would mirror human studies, would also be very compelling. The exploration of pathological activity in other frequency bands, as well as cross-frequency coupling between different bands, may also serve for future reference. Most studies requiring electrophysiological data acquisition performed on rodents, for example, requiring anesthesia, are all dependent on the choice of anesthesia and how that affects the brain [152]. Given this limitation, we would suggest that future studies design such models as to avoid recording under sedation.

Another problem of the studies using anesthetized animals is that electrophysiological recordings were performed during a sleep-like state. We suggest that studies incorporate models equipped to depict at-rest versus task-performing pathological activity, perhaps in a dopaminergic on and off state, perhaps with and without electrical stimulation. For rodents, good tests for evaluating motor deficiency, and its fluctuation under stimulation, would be the rotarod test or the cylinder test. The surgical approach of implantation may be reconsidered in order to resemble more a DBS technique rather than an open cranium surgery.

Stable in vivo long-term electrophysiological recordings are critical in neuroscience, and to this end, a number of technologies have been developed. The use of chronically implanted immobile silicon probes in the primary visual cortex has been demonstrated to provide stable, long-term recordings of neural activity in mice over multiple days [153]. A flexible mesh electronic-based in vivo recording and stimulation platform was developed to address limitations from shear motion and chronic immune responses during long-term recordings [154]. The platform was shown to provide stable multiplexed LFP and single-unit recordings in mouse brains for up to 8 months without the need to reposition the probe [154]. The advanced technology of neuropixels offers high-quality and stable recordings from thousands of sites over prolonged durations (more than 2 months) during free rodent behavior [155]. It would be progressive to see the previously described technologies applied successfully within PD models to study the pathological brain activity.

All of these recommended adjustments would further aid the translational approach.

## Conclusions

We have explored the numerous in vivo electrophysiological hallmarks of PD, among which increased beta oscillations and reduced gamma oscillations characterize the antikinetic state. In the search for novel physiological biomarkers, the study of wave frequencies as well as the use of animal models have proven to be versatile and robust. Despite the limitations of current experimental models in fully replicating the complexities of PD, all of these approaches possess great potential for translation to human studies. The models differentiate not only in the clinical presentation of the disease but also, more importantly, in the histopathological and electrophysiological characteristics, which will significantly affect the results, thus broadening the challenges of translational research. Therefore, mechanistic comprehension behind the animal models used is essential.

To fully comprehend the specific electrophysiological characteristics of PD, a combination of forward and reverse translation is necessary. We addressed the gap between electrophysiological results obtained from studies performed on animal models versus investigations in humans. We intend that this comprehensive picture of the current understanding regarding the pathological activity in PD and animal models of PD provides insights into potential directions for future studies.

## Data Availability

The data that support the findings of Fig. 2 are available upon reasonable request from the corresponding author.
